# Non-Invasive Rheo-MRI Study of Egg Yolk-Stabilized Emulsions: Yield Stress Decay and Protein Release

**DOI:** 10.3390/molecules27103070

**Published:** 2022-05-10

**Authors:** Maria R. Serial, Luben N. Arnaudov, Simeon Stoyanov, Joshua A. Dijksman, Camilla Terenzi, John P. M. van Duynhoven

**Affiliations:** 1 Laboratory of Biophysics, Wageningen University, 6708WE Wageningen, The Netherlands; raquel.serial@tuhh.de (M.R.S.); camilla.terenzi@wur.nl (C.T.); 2 Unilever Global Food Innovation Centre, 6708WE Wageningen, The Netherlands; luben.arnaudov@unilever.com (L.N.A.); simeon.stoyanov@wur.nl (S.S.); 3 Physical Chemistry and Soft Matter, Wageningen University and Research, 6708WE Wageningen, The Netherlands; joshua.dijksman@wur.nl

**Keywords:** enzymatically modified egg yolk, yield stress, high-density emulsions

## Abstract

A comprehensive understanding of the time-dependent flow behavior of concentrated oil-in-water emulsions is of considerable industrial importance. Along with conventional rheology measurements, localized flow and structural information are key to gaining insight into the underlying mechanisms causing time variations upon constant shear. In this work, we study the time-dependent flow behavior of concentrated egg-yolk emulsions with (MEY) or without (EY) enzymatic modification and unravel the effects caused by viscous friction during shear. We observe that prolonged shear leads to irreversible and significant loss of apparent viscosity in both emulsion formulations at a mild shear rate. The latter effect is in fact related to a yield stress decay during constant shearing experiments, as indicated by the local flow curve measurements obtained by rheo-MRI. Concurrently, two-dimensional D-T_2_ NMR measurements revealed a decrease in the T_2_ NMR relaxation time of the aqueous phase, indicating the release of surface-active proteins from the droplet interface towards the continuous water phase. The combination of an increase in droplet diameter and the concomitant loss of proteins aggregates from the droplet interface leads to a slow decrease in yield stress.

## 1. Introduction

Structural stability over time is an essential quality marker for industrial applications of concentrated food emulsions. Throughout processing and shelf life, emulsions are required to maintain constant rheological and microstructural properties for prolonged periods of time, either under static and/or shearing conditions [1,2]. Yet, food emulsions are inherently thermodynamically unstable, and special care must be taken to overcome their inherent tendency to destabilize or break. Egg yolk (EY), which is an important source of phospholipids and proteins with emulsifying properties, has been widely used as a natural emulsifier in foods, such as mayonnaise and salad dressings [3]. EY emulsifying capacity is, however, highly dependent on conditions, such as temperature, pH and ionic strength [4,5]. The latter severely limits the design of egg yolk-based formulations in the food industry and current research has been focused on developing ways to improve their thermal stability and emulsifier capacity.

Enzymatic treatment with phospholipase A_2_ (PLA_2_) is a very effective method used in industry to increase EY functionality. PLA_2_ cuts off the acyl group position 2 of the triglyceride and converts the phospholipids into lyso-phospholipids, improving the emulsifying properties in oil-in-water emulsions [4,6,7]. Recently, Gazolu-Rusanova et al. [7] proved that the interfacial properties of enzymatically modified egg yolk (MEY) are governed by the presence of lyso-phospholipids and oleic acid formed during enzymatic conversion, which in turn increases the interfacial tension and improves the emulsion film properties. However, up to now, little has been reported about the rheological properties of food emulsions prepared using MEY as an emulsifier.

The rheological properties of EY-stabilized emulsions have been, on the contrary, thoroughly studied in the literature, in terms of their time-dependent yield stress behavior [8,9,10,11,12,13]. Such observations have been reported for different droplet sizes and concentrations, as well as for a wide range of applied shear rates. In most studies, droplet flocculation dynamics and/or breakage of cluster bonds are mentioned as possible explanations for the observed time-dependent behavior in EY emulsions. Yet, most papers either do not provide enough experimental evidence to corroborate such hypotheses, or they focus only on a macroscopic rheological characterization, which in turn cannot infer shear-induced changes at the microstructural level [14,15].

Here, we investigate the time-dependent behavior of EY- and MEY-stabilized concentrated emulsions during measurements at constant shearing. In order to obtain in situ local flow curves, we combined the results from conventional rheology with those from localized flow measurements performed by rheo-MRI. This approach was successfully validated for the quantitative assessment of time-dependent yield stress behavior in complex systems, such as micronized fat crystals dispersions undergoing structural degradation [16]. The results shown in the present work indicate that EY and MEY emulsion formulations exhibit a decay of the apparent viscosity under constant shear, caused by an irreversible decrease of the emulsion’s yield stress value. Furthermore, shear-induced microstructural changes in the water phase of the emulsions are assessed by two-dimensional ^1^H diffusion-T_2_ (D-T_2_) NMR correlation measurements [17,18,19,20,21]. We show that shear induces an increase in oil droplet size and rearrangement of proteins between the droplet interface and water phase for both EY and MEY emulsion formulations, explaining the corresponding decay of yield stress.

## 2. Results

### 2.1. Impact of Shear on the Rheological Properties

**Figure 1:**
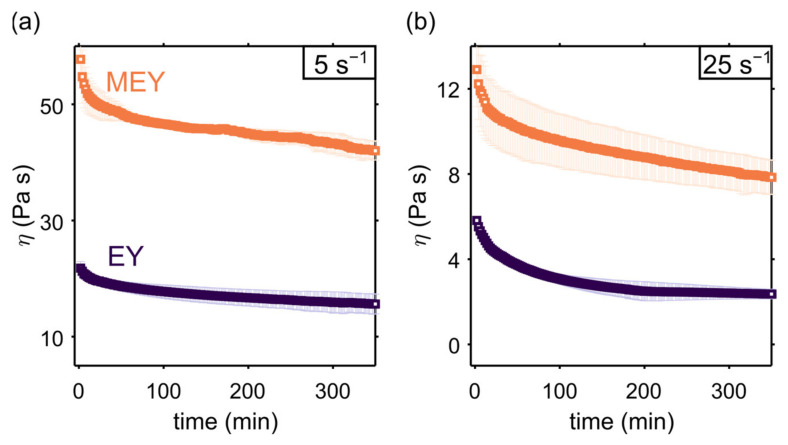
Apparent viscosity measurements of EY (dark purple) and MEY (orange) emulsion formulations as a function of time, performed at a constant shear rate of 5 s^−1^ (**a**) and 25 s^−1^ (**b**) using a Couette cell geometry (gap size 1 mm). Symbols refer to means over two replicates, and error bars represent the deviation range.

We investigate the time-dependent behavior of EY and MEY dense emulsions by recording apparent viscosity values at two different applied shear rates (5 and 25 s^−1^). As shown in Figure 1, the MEY emulsion displays a significantly higher apparent viscosity at both shear rates, compared to the non-modified EY emulsion. Recently, Gazulu-Rosanova et al. thoroughly studied the interfacial properties of native EY and MEY, showing that, under acidic conditions, the enzymatic treatment led to a considerably higher interfacial tension, caused by the presence of lyso-phospholipids and oleic acids [7]. Since the EY and MEY emulsions studied in the present work differ from those examined in [7], only with respect to the emulsifier used, the resulting differences in apparent viscosity suggest that interfacial tension dominates the stress response in both emulsions. From the results shown in Figure 1, it is also evident that both formulations are sensitive to shear treatment, exhibiting a time-dependent viscosity decay. A similar behavior was also found for mayonnaise and other similar food products by several authors [8,9,10,13,22]. Surprisingly, both EY and MEY emulsion formulations did not reach a constant viscosity value in the time range studied, up to ∼500 min.

**Figure 2:**
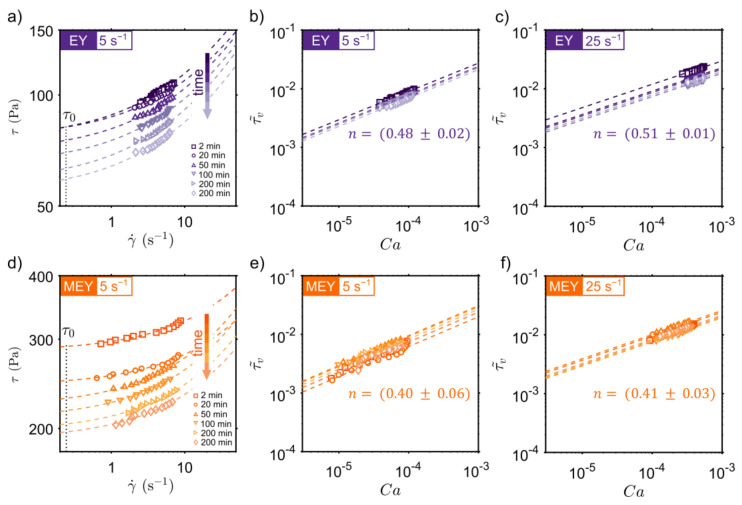
Time dependence of (**a**,**d**) local flow curves, $\tau \left(\dot{\gamma }\left(r\right)\right)$, and of (**b**,**c**) or (**e**,**f**) dimensionless viscous stress, ${\tilde{\tau }}_{v}$, during shear, respectively, at 5 s^−1^ and 25 s^−1^, for EY (**a**–**c**) and MEY (**d**–**f**) emulsion formulations. Dotted lines in all plots correspond to a Herschel–Bulkley fitting.

**Figure A1:**
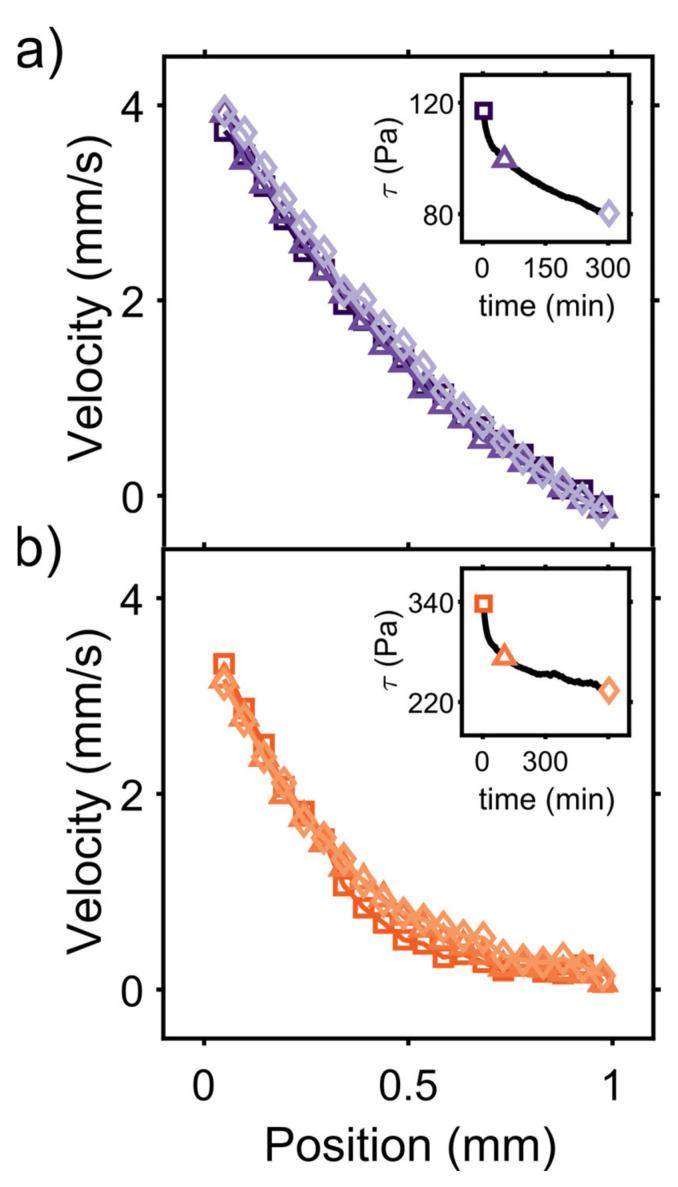
^1^H rheo-MRI velocity profiles of (**a**) EY and (**b**) MEY emulsions at different times during shearing experiments at 5 s^−1^: 2 min (squares), 50 min (triangles) and 300 min (diamonds). Insets show the associated stress values at each time.

To go deeper into the analysis of the time-dependent behavior of EY and MEY emulsions, conventional rheology measurements are combined with localized 1D flow profiles recorded using rheo-MRI. This technique has been extensively applied in the study of structure formation and degradation of complex systems, as well as in the investigation of transient flow in Newtonian and non-Newtonian fluids [16,23,24,25]. By combining rheology stress (σ) measurements with shear rate $\dot{\gamma }$(r) profiles calculated from rheo-MRI velocity data, with r being the position along the cell gap, a local flow curve (LFC) $\sigma \left(\dot{\gamma }\left(r\right)\right)$ can be constructed as a function of time during shear treatment. Figure 2 displays the time evolution of LFCs obtained for EY (top) and MEY (bottom) emulsions, recorded at different times under a constant applied shear rate. Obtained results revealed structural degradation as the LFCs shifted to lower shear stress values. It is worthwhile noting that MEY LFCs show a wider shear rate dispersion compared to EY formulation due to the presence of shear localization in MEY velocity profiles (See Figure A1 in Appendix A).

LFCs in Figure 2 can be well fitted using a simple Herschel–Bulkley (HB) model (dashed lines) given by: $\tau =\tau_{0}+\tau_{v}\left(\dot{\gamma }\right)=\tau_{0}+K\;{\dot{\gamma }}^{n}$, where $\tau_{0}$ is the yield stress of the emulsion and $\tau_{v}$ is the viscous stress that depends on the shear rate $\dot{\gamma }$, consistency number K, and the power-law index n. In all cases, n was taken as a global fitting parameter. A useful approach to compare emulsions prepared using different surfactants is to plot the viscous stress normalized to the emulsion’s average droplet size radius (R) and interfacial tension (σ) versus the capillary number $Ca=\mu \;\dot{\gamma }R/\sigma$. The time evolution of the normalized viscous stress $\tilde{\tau_{v}}=\tau_{v}R/\sigma$ is displayed in Figure 2b,c for EY and Figure 2e,f for the MEY emulsions. We found that for both EY and MEY formulations, the dimensionless viscous stress $\tilde{\tau_{v}}$ does not appreciably change during shear treatment at 5 s^−1^ and 25 s^−1^. In fact, both emulsion formulations exhibited a flow index n∼0.47 for the entire shearing time studied, as expected for emulsions with high volume fractions [26,27,28]. This suggests that the main cause of the decrease in apparent viscosity is a temporal decay in the emulsion’s yield stress.

**Figure 3:**
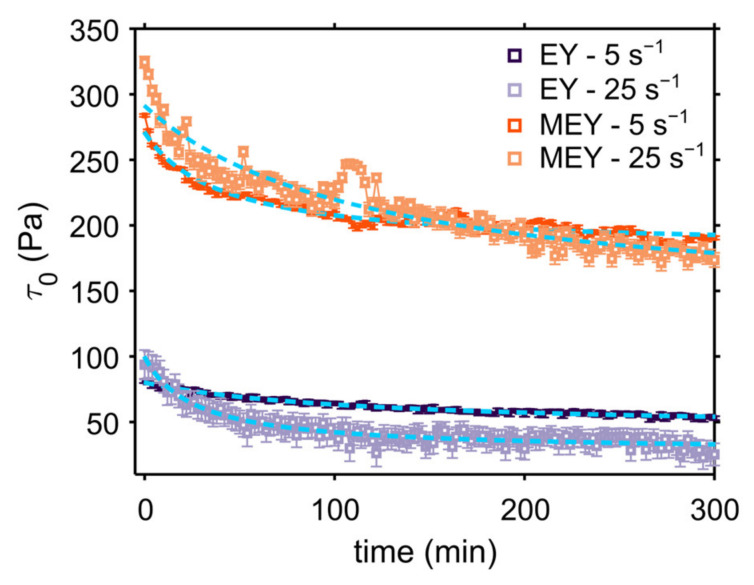
Yield stress values of EY and MEY emulsions as a function of shearing time at 5 s^−1^ and 25 s^−1^ and SKM fitting curves (dashed blue lines). Plotted error bars correspond a Herschel- Bulkley fitting error and refer to a single experiment. For each formulation, the relative standard deviation errors for duplicates are in the order of 10 %.

Figure 3 shows the fitted $\tau_{0}$-values as a function of time for both formulations during shearing measurements at 5 and 25 s^−1^. As expected, MEY emulsion exhibits a significantly higher yield stress value compared to non-modified EY emulsion. The scaling difference between EY and MEY emulsions can be easily explained by the expected proportionality of $\tau_{0}$ with the equilibrium interfacial tension, σ, and the average droplet size distribution at high volume fractions of ϕ>80% [29,30,31,32]. As suggested by the LFC data, the yield stress of both emulsions also decays as a function of shearing time, indicating shear-induced structural degradation.

Several models have been proposed in the literature to describe the time-dependent behavior of complex materials upon constant shearing. In particular, the second-order structural kinetic model (SKM) has been successfully applied to study the time dependence of several food suspensions, emulsions and pastes [9,13,33,34,35]. For example, Nguyen et al. used the SKM to study the time-dependent yield stress behavior of waxy maize starch pastes [33]. This model assumes that changes in the rheological properties upon shearing are related to a breakdown of the internal structure. Therefore, the rate of structural breakdown will depend on the kinetics of the system, transitioning from its initial yield stress value $\tau_{0,0}$ (structured state) towards the final, or equilibrium, state $\tau_{0,e}$. In this way, we can define the time dependence of the yield stress, $\tau_{0}\left(t\right),$ as follows [33]:

(1) $$\tau_{0}\left(t\right)=(\tau_{0,0}-\tau_{0,e}){\left(k_{s}t+1\right)}^{-1}+\tau_{0,e}$$

In the latter equation, $\tau_{0}\left(t\right)$ is the yield stress value as a function of shearing time t, and $k_{s}$ is the rate of the structural breakdown. By fitting the yield stress decay, the rate and extent of structural breakdown can be quantified for native EY and MEY emulsions under shear. The obtained fitting parameters are presented in Table 1, and show that, at 5 s^−1^, the structural breakdown rate, $k_{s}$, is by an order of magnitude lower for the EY emulsion compared to the MEY emulsion. At higher shear rates, however, both emulsions show similar $k_{s}$ values. These values are in line with the ones reported by Tiu and Borger et al. for commercial mayonnaise [8].

On the other hand, the ratio between the initial and equilibrium yield stress values $\tau_{0,0}/\tau_{0,e}$, which gives information on the extent of structural breakdown, is higher for the EY emulsion, corroborating that the MEY emulsion is more resistant to shear treatment. Comparable results were obtained by Abu-Jdayil et al. in the study of model egg-yolk mayonnaise (oil concentration between 10–64%) [13].

### 2.2. Effect of Constant Shear on Microstructure and Protein Distribution

Rheology and rheo-MRI results showed that shear causes structural degradation triggered by a time decay of the emulsion yield stress value. We now look at the effect of shear on the emulsion’s microstructure.

**Figure A2:**
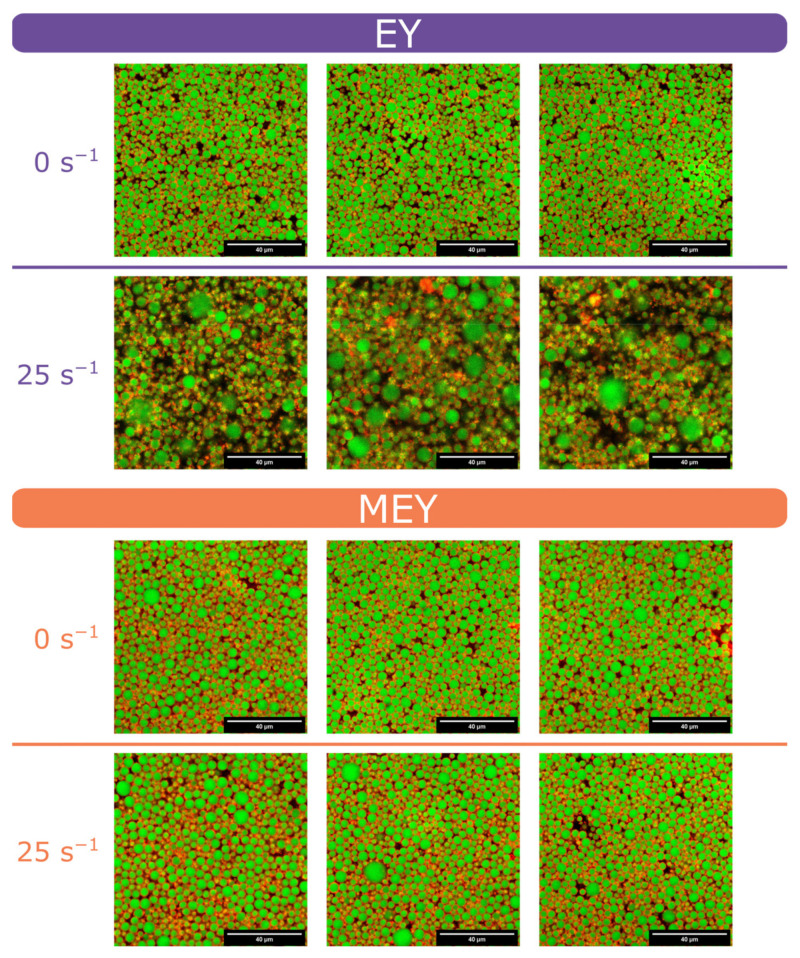
Representative CLSM images of 80% diluted EY (top) and MEY (bottom) emulsions before (0 s^−1^) and after shearing treatment at 25 s^−1^ for 300 min.

In the literature, irreversible structural breakdown in emulsions is often associated with the coalescence of droplets. To check this behavior in our experiments, we analyzed the droplet size distribution before and after shearing at 5 s^−1^ and 25 s^−1^ by PFG NMR measurements [36]. The obtained results are summarized in Table 2. Before shear, the emulsions prepared with EY and MEY have similar droplet size distributions (D_3,3_, α), in agreement with the confocal imaging experiments (CLSM) shown in Figure A2. After shearing, the EY emulsion is more susceptible to coalescence than MEY, displaying a considerable increase in droplet size, especially at 25 s^−1^ (D_3,3_/D_3,3(0)_ = 2.33). On the contrary, the MEY emulsion proves to be much more robust to shear treatment, exhibiting only a slight increase (D_3,3_/D_3,3(0)_ = 1.26) in droplet size at 25 s^−1^. The latter behavior can be further corroborated by observing the CLSM images acquired before and after shearing measurements in Figure A2, where the EY emulsion’s microstructure is remarkably changed by shear, showing signs of strong coalescence and droplet aggregation.

**Figure 4:**
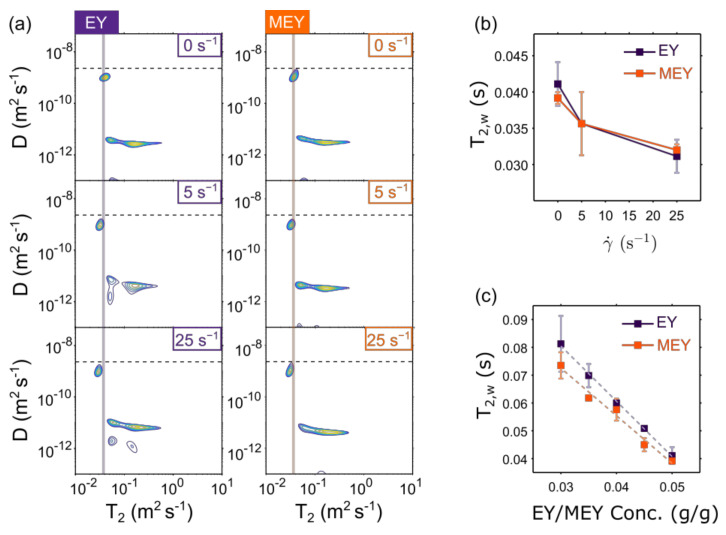
(**a**) ^1^H D-T_2_ NMR correlation maps of EY (left) and MEY (right) emulsion formulations before shearing (top) or after shearing at 5 s^−1^ and 25 s^−1^ for 300 min. The horizontal dotted line in all plots corresponds to the diffusion coefficient of free water at 25 °C. The vertical solid lines in the left and right plots (respectively, in dark purple and orange) correspond to the initial T_2,w_ value before measurements at constant shear. The T_2, w_ values of EY and MEY emulsions as a function of (**b**) the applied constant shear rate $\dot{\gamma }$ and (**c**) the concentration of EY/MEY samples at varying dilutions in water. Symbols in plots (**b**,**c**) are means over two replicates, and error bars are their respective standard deviations.

We also investigated compositional changes in EY and MEY emulsions before and after shearing by measuring ^1^H diffusion-T_2_ (D-T_2_) NMR correlation maps. This technique allows us to separate the signal contribution of the aqueous phase from the oil phase. Figure 4a displays acquired D-T_2_ correlation maps for EY and MEY emulsions before (0 s^−1^) and after shearing treatment at 5 s^−1^ and 25 s^−1^. In all D-T_2_ distributions, mainly two components are observed. The one with the highest diffusion coefficient ($D∼9\times 10^{-10}$ m^2^ s^−1^) is close to the diffusion value expected for free water (dashed line) and is therefore associated with the emulsion’s aqueous phase. The second component displays a wide range of T_2_ NMR relaxation times with a signal distribution around a lower diffusion coefficient value, namely $D∼4\times 10^{-12}$ m^2^ s^−1^. The latter component was associated with the oil phase of the emulsion. These population assignments are in line with the D-T_2_ correlation results obtained for other oil-in-water food emulsions, such as cream and soft or hard cheeses [18,21].

It is important to point out that the 2D inverse Laplace transformation (ILT) used to obtain the D-T_2_ correlation maps in Figure 4a assumes exponential behavior for both encoding dimensions. This is indeed the case for the aqueous phase signal. However, the non-exponential restricted diffusion behavior of the oil phase cannot be handled by the 2D ILT algorithms, which yield spurious signals in the D-T_2_ map. As a consequence, oil phase D-T_2_ signals cannot be used for the quantitative assessment of shear-induced microstructural changes. Therefore, in this work, the D-T_2_ data have only been used to assess the behavior of the aqueous phase upon shear treatment.

One striking observation from Figure 4a is that shear causes a decrease in the T_2_ relaxation time of the aqueous phase (T_2,w_). As shown in Figure 4b, T_2,w_ decreases with applied shear, showing a similar trend for both EY and MEY emulsion formulations.

We note that in our experiments, the water diffusion length ($\sqrt{2D_{w}\Delta }\approx$ 10 μm) is of the same order of magnitude as the emulsion’s droplet size (Table 2). Varying the ∆-values did not result in significant changes in the D-T_2_ correlation maps. Similar findings were reported in the study of systems with similar microstructural length scales and employing comparable parameters [18,21].

In dairy products, several authors have shown that T_2,w_ depends mainly on the concentration of dissolved proteins [18,21]. To verify this for our EY and MEY emulsions, we performed dilution experiments. In Figure 4c T_2,w_ is plotted against the protein concentration in EY/MEY, showing a linear decrease for both formulations. From this, we can infer that the T_2,w_ decreases after shearing treatment is due to an increase in protein concentration in the water phase of the EY/MEY emulsions.

## 3. Discussion

### 3.1. Impact of Enzymatic Treatment of Egg Yolk

The difference in the rheological behavior of EY and MEY emulsions can be explained in terms of the impact of the enzymatic treatment on the interfacial properties of egg yolk. Besides converting the phospholipids to more surface-active lyso-phospholipids, the enzymatic reaction alters the structure of lipoprotein particles and also the interfacial tension properties. Recently, Gazulu-Rosanova et al. thoroughly studied the interfacial properties of native EY and MEY, finding an important increase in interfacial tension properties of enzymatically modified egg yolk under acid conditions [7]. This behavior was attributed to the breaking of protein granules during enzymatic treatment, releasing lyso-phospholipids and oleic acid agents, which in turn dominate the interfacial properties of MEY. For the particular case of emulsions with a high dispersed volume fraction ∅, the yield stress $\tau_{0}$ is expected to depend on droplet elasticity, which scales as the ratio between interfacial tension σ and the average droplet size D3.3 [27,32,37]. Since our EY and MEY emulsions have similar droplet size distributions, our results agree with the work done by Gazulu-Rosanova et al., not only showing an improved performance of MEY as an emulsifier but also implying that interfacial tension determines the yield stress of both emulsions.

### 3.2. Impact of Low Shear Treatment

**Figure 5:**
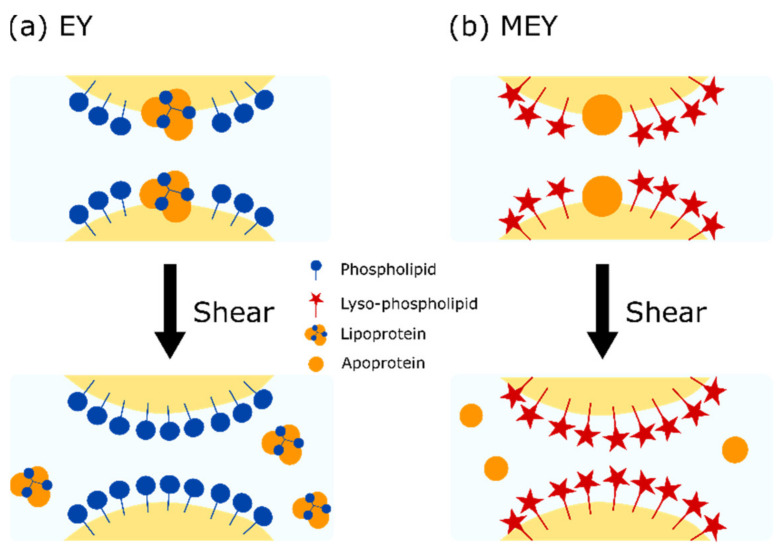
Schematic representation of the effect of enzymatic modification and shear treatment on surface composition of (**a**) EY and (**b**) MEY emulsions.

The conversion of phospholipids to the more surface-active lyso-phospholipids in MEY causes less droplet coalescence upon mild shear (5 and 25 s^−1^), as can be seen in Table 2. The mild shear treatment leads to a significant decrease in the yield stress for both EY and EMEY emulsions. In the case of a constant σ, the ratio $\frac{\tau_{0,0}}{\tau_{0,e}}\;$ should be proportional to $R_{e}/R_{0}$, where $R_{0}$ and $R_{e}$ are the average droplet radius before and after shearing, respectively. Table 1 shows the comparison between both quantities, showing that droplet size variations explain a major part of the observed changes in the emulsion yield stress. For the remaining effect, we consider the increase in protein concentration in the continuous phase, as indicated by D-T_2_ measurements. This implies that apoproteins and lipoprotein particles are released from the interface between droplets, schematically depicted in Figure 5. In steadily sheared emulsions, the film thickness between the droplets increases, while the volume of the Gibbs–Plateau borders decreases [27,28]. Detachment of protein aggregates from the droplet’s interfaces and their transport to the Gibbs–Plateau borders could explain the observed change in the D-T_2_ values. This loss of protein aggregates from the interface can lead to less attractive interactions between oil droplets, which will contribute to lower yield stress.

## 4. Materials and Methods

### 4.1. Preparation of EY and MEY Emulsions

Emulsions were prepared by emulsification using a Silverson mixer (Papendrecht, The Netherlands). Two formulations were made employing normal egg yolk (EY) and egg yolk modified by enzymatic treatment (MEY) blends as emulsifiers, containing 8% (*w*/*w*) NaCl. In both cases, the aqueous phase was prepared first, composed of EY or MEY blend (5% *w*/*w*) and NaCl (0.7% *w*/*w*) dissolved in distilled water (14.8% *w*/*w*). Finally, rapeseed oil (78% *w*/*w*) was slowly added and mixed at 8500 rpm for 4 min to form the emulsion. Spirit vinegar (1.5% *w*/*w*) was added and mixed for a further 2 min.

Rapeseed oil was obtained from ADM (Rotterdam, Netherlands). Non-modified egg yolk and egg yolk modified by enzymatic treatment with phospholipase A2 (PLA2) were supplied by Bouwhuis Enthoven (Raalte, The Netherlands), which contained 8% sodium chloride, and were stored at 4 °C.

### 4.2. Rheo-MRI Measurements

Rheo-MRI experiments were performed on a Bruker Avance III spectrometer, operating at a resonance frequency of 300 MHz for ^1^H (7 T). Excitation and detection of the ^1^H signal were performed using a 25 mm inner diameter birdcage coil in combination with a 3D gradient system with a maximum gradient of 1.5 Tm^−1^. To perform strain-controlled experiments during MRI measurements, a standard Bruker rheo-MRI accessory was used and equipped with a homebuilt Couette cell (CC) geometry, with inner and outer diameters of 20 mm and 22 mm, respectively, resulting in a gap size of 1 mm.

Velocity profiles were measured by employing a pulsed gradient spin-echo (PGSE) sequence (velocity encoded gradients duration (δ) of 1 ms and time separation (Δ) of 13.1 ms). For all experiments, a 1 mm × 1 mm slice was excited and locally averaged velocities within the excited slice were measured with a field of view of 25 mm with a spatial resolution of 48.8 µm. To avoid chemical-shift artifacts on the acquired profiles, a CHESS module was applied just before the PGSE sequence, as implemented by Nikolaeva et al. [16]. For all measurements, the signal was averaged over 16 acquisitions, resulting in a total duration of 2 min per experiment.

Shear-controlled experiments were performed simultaneously in a conventional rheometer and in the rheo-MRI setup, employing the same Couette cell (CC) geometry. Velocity profiles were used to deduce the shear rate variation across the cell gap ($\dot{\gamma }$(r)) according to the equation $\dot{\gamma }\left(r\right)=\;r\;\frac{\partial v\left(r\right)}{\partial r}$. The local stress $\sigma \left(r\right)$ was estimated from torque (T) measurements as $\sigma \left(r\right)=\frac{T}{2{\pi r}^{2}H}$ with H being the height of the cell. Local flow curves (LFC) were then obtained by combining local stress $\sigma \left(r\right)$ and shear rate $\dot{\gamma }$(r) information. All experiments were conducted in duplicate.

All calculations and corrections of the rheo-microMRI velocity profiles, as well as the determination of local shear rates and stresses, were performed in Matlab-R2021b (MathWorks). A Savitzky–Golay (SG) FIR smoothing filter was used to obtain the first derivative of the velocity data and calculate the shear rate variations as a function of position in the gap [16,38]. For all experiments, a first-order polynomial fit was used with a window length set of 7 points.

### 4.3. Rheology Experiments

Rheological measurements were conducted on a conventional Modular Compact Rheometer 301 (MCR301, Anton Paar, Graz, Austria). For all experiments, a home built 1 mm gap Couette cell (CC) with the exact same dimensions as the cell used for rheo-MRI experiments was employed [16,25]. Time-dependent measurements of native EY and MEY emulsions were investigated for a total period of 500 min at 20 °C, in synchronization with rheo-MRI experiments. A fresh sample was loaded into the measuring system before every experiment. Measurements were performed at a constant shear rate with two replicates per shear rate value.

### 4.4. Confocal Microscopy

Approximately 2 grams of the emulsion was stained with a droplet of Nile Blue (1% *w*/*w* aq). The stained mayonnaise samples were placed on a glass slide that was plasma cleaned for 90 s to avoid electrostatic attraction and subsequent coalescence of oil droplets. Samples were imaged with a Zeiss LSM880 confocal scanning laser microscope in combination with an Axio Observer Z1 inverted microscope. The specific coloring capabilities of Nile Blue for oil and proteins were detected in two tracks, respectively, with excitation at 488 nm, emission at 520–670 (displayed in green) and excitation at 633 nm, emission at 660–750 nm (displayed in red).

### 4.5. D-T_2_ Measurements

^1^H D-T_2_ NMR experiments were performed on a Bruker Avance III spectrometer equipped with a diff25 probe, operating at a resonance frequency of 300 MHz for ^1^H (7 T). Correlation maps were measured using a stimulated echo-based PGSE diffusion pulse sequence in combination with unipolar, trapezoid-shaped gradient pulses For the T_2_ dimension, a CPMG (Carr–Purcell–Meiboom–Gill [39,40]) sequence was used, with detection of 256 echoes and an echo spacing of $t_{E}=2.2$ ms. In all experiments, an effective diffusion time (Δ) of 200 ms and an effective gradient pulse width (δ) of 1 ms was used. Gradient strength was varied in a linear manner between 0.1–6.6 Tm^−1^ in 32 steps. The NMR signal was averaged 8 times with a repetition time of 4 s. In all cases, measurements were performed in duplicate.

### 4.6. Droplet Size Measurements

Oil droplet size measurement was performed by means of pulsed-field gradient (PFG) NMR using a time-domain Minispec mq20 spectrometer (Bruker, Rheinstetten, Germany) operating at 20 MHz for ^1^H (0.47 T). In the pulse sequence, the gradient strength was varied in order to obtain PFG diffusion decays [41]. A diffusion time (Δ) of 211 ms and a measurement temperature of 20 °C were used. The intra-droplet restricted self-diffusion of oil protons can be described by a modified Murday–Cotts equation [42,43]. Fitting of PFG diffusion decays using this equation allows to obtain the droplet size distribution, expressed as the volume-weighted average D_3.3_ and width of the lognormal distribution α.

## 5. Conclusions

Rheo-MRI can be used to assess local flow curves during mild shear treatments. This allows for quantitative kinetic assessment of the yield stress of EY and MEY stabilities during mild shear treatment. The MEY-stabilized emulsions are much more stable to shear treatment due to the conversion of phospholipids to stronger emulsifying lyso-phospholipids. For both EY- and MEY-stabilized emulsions, droplet sizes increase upon mild shear treatment, which explains a major part of the loss of yield stress. The loss of apoproteins and/or lipoprotein particles adsorbed at the droplet interface can lead to a loss of surface tension and decreased adhesive forces, both contributing to a loss of yield stress.

## Figures and Tables

**Table 1:** Fitting parameters obtained from the SKM analysis of rheological data for EY and MEY emulsions, before and after shearing at 5 s^−1^ and 25 s^−1^.

	$\dot{\gamma }\;\left(s^{-1}\right)$	$k_{s}\;(s^{-1})$	$\tau_{0,0}\;(\mathrm{Pa})$	$\tau_{0,e}\;(\mathrm{Pa})$	$\tau_{0,0}/\tau_{0,e}$
EY	0 *	-	-	-	-
	5	0.012 ± 0.001	80.1 ± 0.5	44 ± 1	1.82 ± 0.01
	25	0.06 ± 0.01	99 ± 2	27 ± 1	3.66 ± 0.01
MEY	0 *	-	-	-	-
	5	0.11 ± 0.01	271 ± 2	182 ± 1	1.49 ± 0.01
	25	0.04 ± 0.01	291 ± 4	134 ± 7	2.17 ± 0.03

* corresponds to samples before shearing treatment.

**Table 2:** Droplet size distribution measurements (mean diameter d_3,3_ and distribution width α) of EY and MEY emulsions before and after shearing at 5 s^−1^ and 25 s^−1^.

	$\dot{\gamma }\;\left(s^{-1}\right)$	$D_{3,3}\;(\mu m)$	α (μm)	D_3,3_/D_3,3(0)_
EY	0 *	3.39 ± 0.01	0.20 ± 0.01	-
	5	4.30 ± 0.01	0.40 ± 0.01	1.27 ± 0.01
	25	7.88 ± 0.02	0.763 ± 0.004	2.33 ± 0.01
MEY	0 *	3.84 ± 0.01	0.30 ± 0.01	-
	5	4.30 ± 0.01	0.30 ± 0.01	1.12 ± 0.01
	25	4.85 ± 0.01	0.35 ± 0.01	1.26 ± 0.01

* corresponds to samples before shearing treatment.

## Data Availability

Supporting data can be found at Zenodo, https://doi.org/10.5281/zenodo.6491052.
